# A Critical Review of Emerging Solutions for Food Packaging: Opportunities and Challenges

**DOI:** 10.3390/foods15050920

**Published:** 2026-03-06

**Authors:** Joana C. L. Martins, Juliana Garcia, Rafaela Guimarães, Irene Gouvinhas, Maria José Alves, Maria José Saavedra

**Affiliations:** 1 Centre for Water Technology Valorisation and Transference, AquaValor, 5400-342 Chaves, Portugal; 2 Centre for the Research and Technology of Agroenvironmental and Biological Sciences, CITAB, Inov4Agro, University of Trás-os-Montes and Alto Douro (UTAD), Quinta de Prados, 5000-801 Vila Real, Portugal; 3 Animal and Veterinary Research Center (CECAV), University of Trás-os-Montes and Alto Douro (UTAD), 5000-801 Vila Real, Portugal; 4 Research Centre for Active Living & Wellbeing (LiveWell), Polytechnic Institute of Bragança, 5300-253 Bragança, Portugal; 5 Mountain Research Centre (CIMO), Polytechnic Institute of Bragança, Santa Apolónia Campus, 5300-253 Bragança, Portugal; 6 Associated Laboratory for Sustainability and Technology in Mountain Regions (SusTEC), Polytechnic Institute of Bragança, Santa Apolónia Campus, 5300-253 Bragança, Portugal; 7 Associate Laboratory of Animal and Veterinary Sciences (AL4AnimalS), Faculty of Veterinary Medicine, Avenida da Universidade Técnica, 1300-477 Lisboa, Portugal

**Keywords:** food packaging, active packaging, biopolymers, bioactive compounds, sustainability

## Abstract

The environmental impact of conventional plastics has driven a shift toward biobased food packaging, shaped by consumer expectations, market trends, and regulatory policies within the European Union (EU). Despite extensive research on biopolymers such as starch, cellulose, chitosan, and polylactic acid (PLA), their use in commercial food packaging remains limited. A major challenge identified in the literature is the evaluation of biopolymer performance, in which environmental benefits are often considered independently of mechanical, barrier, and economic factors. This review addresses this gap by critically exploring the functional performance of biopolymers regarding their chemical structure and processing methods, with particular emphasis on the role of bioactive compounds in enhancing these materials’ properties. Although several biopolymers can achieve tensile strength values comparable to conventional petroleum-based plastics, their higher water vapor transmission rates remain an unsolved barrier to scalability. These limitations, together with challenges related to mechanical performance and production costs, are discussed to clarify their impact on industrial feasibility and to identify priorities for future research supporting scalable, cost-effective, and regulatory-compliant food packaging solutions.

## 1. Introduction

Every year, more than 400 million tonnes of plastic are produced worldwide, of which around 36% is used for packaging, including single-use plastics for food and beverages; only 25% is recycled [1,2]. There is an urgent need to rethink the packaging sector through sustainable strategies. However, these strategies do not depend exclusively on material replacement and extend beyond environmental indicators to include social, economic, and functional factors [3]. A comprehensive approach is needed to assess packaging performance in the broader context of circular economy principles, food preservation efficiency, and consumer acceptance.

Despite the progress in biobased food packaging, there are still gaps in research across different scopes. From a materials perspective, biobased and biodegradable alternatives continue to face challenges related to mechanical performance, barrier properties, and scalability [4,5]. Regarding the safety standpoint, concerns remain considering the migration and toxicological effects of new materials, including nanoparticles, (per- and polyfluoroalkyl substances) PFAS, and microplastics [6,7]. At the consumer level, there is a duality between the growing desire for sustainable packaging alternatives and the demand for affordability, leading to a discrepancy between consumer intentions and actual purchasing behaviour, as well as the need for market-specific strategies [8,9]. Finally, within the policy and assessment perspective, methodological inconsistencies in lifecycle assessment (LCA) and insufficient end-of-life infrastructure limit the accurate sustainability evaluation, while evolving regulatory frameworks delay harmonisation and weaken consumer trust [10,11]. Addressing these research gaps separately has limited the development of viable strategies for sustainable packaging solutions, underscoring the need for an interdisciplinary approach that connects materials science, safety assessment, consumer behaviour, and regulatory policy. In this context, the present manuscript focuses on the shift from conventional petroleum-based plastics to bioactive and biodegradable food packaging systems. It critically explores biopolymers, such as starch, cellulose, and chitosan, as packaging matrices and bioactive compounds (including polyphenols and terpenoids) extracted from agri-food waste and by-products. This narrative review aims to establish a multidimensional framework that synthesises recent developments, identifies key limitations, and highlights future challenges for sustainable food packaging innovation.

The literature analysed was identified through comprehensive searches of major scientific databases, including Web of Science and Scopus. Peer-reviewed publications from the last decade were prioritised, using keywords including combinations of biobased and biodegradable packaging, bioactive materials, food-contact safety, barrier properties, consumer behaviour, and regulatory frameworks. Additional relevant studies were identified through citation tracking of key publications.

## 2. Food Packaging

**Figure 1:**
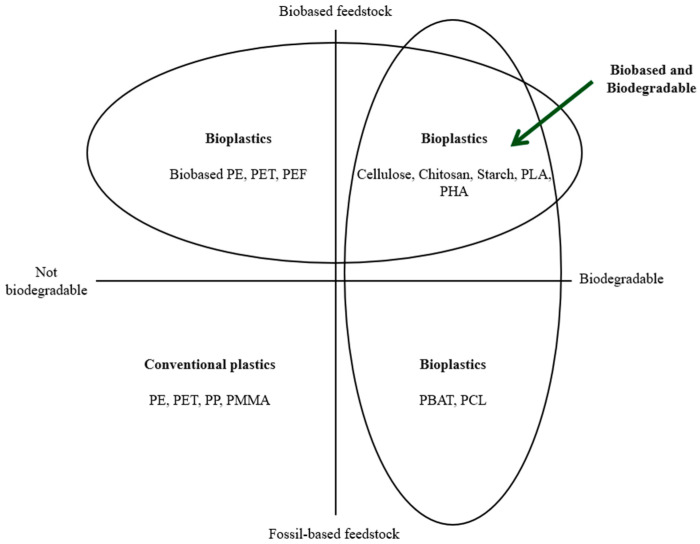
Plastic materials characterisation on origin and biodegradability.

Floros et al. [12] defined food packaging as “a complex and dynamic system aiming to safely prepare foods for transportation, distribution, storage, retailing, handling, and end-use, and safely deliver these foods to the consumer in a sound condition (maximum quality) at a minimum cost”. The importance of food packaging is directly related to the safety and quality of food products throughout the entire supply chain, “from farm to fork” [13]. The primary role of packaging is to protect food products from external factors and maintain their integrity. Additionally, food packaging is a marketing tool to attract consumers and provide valuable information, such as ingredients, nutritional information, and traceability information [13,14]. While food packaging is fundamental for ensuring food safety and security, it also presents several challenges. These include the need for environmentally friendly packaging materials, compliance with regulations and guidelines to ensure food safety, and addressing consumer demands for convenience and ease of use while minimizing waste [7,14]. The growing environmental awareness in Europe prompted the European Commission (EC) to present the ongoing plan to replace fossil-based feedstock plastics with biobased materials. Figure 1 summarizes the origin and biodegradability of the materials used in the packaging sector.

**Figure 2:**
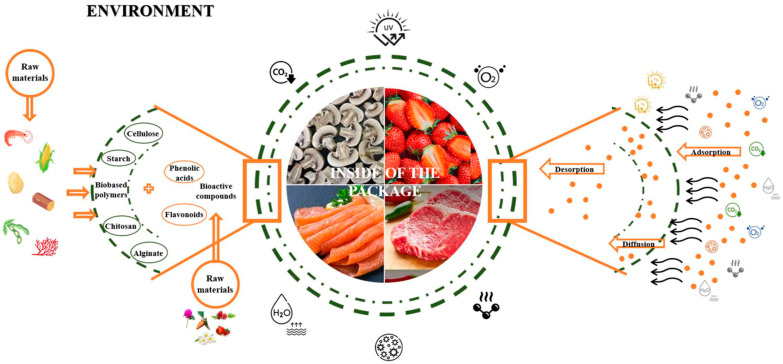
Emerging sustainable alternatives and environmental interactions of food packaging.

One of the eco-friendly solutions that emerged was biobased and biodegradable bioplastics [15]. In line with this, agri-food waste and by-products can be a source of these materials (fibres and other polysaccharides) for food coatings, trays, containers, disposable packaging, and biobased polymer packaging. To be considered biodegradable, these bioplastics must initially decompose into smaller molecules, such as H_2_O, CO_2_, and CH_4_, in anaerobic conditions, which are then absorbed and digested by microbes, forming new microbial biomass and completing the biodegradation process [16,17]. Moreover, these materials must exhibit specific characteristics that ensure the preservation and quality of the packaged products. These include the ability to facilitate controlled oxygen transference, serve as a selective barrier to carbon dioxide and moisture, prevent lipid migration, improve structural integrity, and minimize the loss of volatile biological components, thus easing the mechanical handling of foods. These mutual interactions between the environment and the packaging and, consequently, the food product can be explained by the sorption processes (absorption and desorption), and the diffusion mechanisms; both are important to guarantee the performance and effectiveness of food packaging materials [18,19,20]. On the one hand, absorption refers to the retention of substances from the environment within the packaging material, whereas desorption is the release of these substances from the material into the package interior, directly influencing food quality and shelf-life [18,19,20]. The principal models used to describe the sorption mechanisms are Henry’s, Langmuir’s, and Flory–Huggins’s models [21]. The diffusion mechanism, described using Fick’s laws [22], consists of the transference of molecules through the packaging material and is determined by factors such as the molecular size of the substances, the composition and structure of the packaging material, and environmental conditions [18,19]. Figure 2 summarizes the emerging sustainable alternatives for packaging materials and the environmental interactions of food, packaging, and the environment. Additionally, these packaging materials should incorporate bioactive compounds, such as antimicrobials and antioxidants, to provide a protective barrier against spoilage microbes [23]. This type of packaging, known as active packaging, involves integrating bioactive compounds into the polymer to enhance its effectiveness by protecting food against deterioration, extending shelf life, and inhibiting microbial growth, which is frequently linked to food loss and waste [24]. As bio-compounds, natural extracts from agri-food waste and by-products have been shown to improve packaging properties, such as physical and mechanical characteristics, while potentially offering antioxidant and antimicrobial effects, extending food shelf life, and reducing dependence on synthetic additives [25]. This adds value to food industry waste streams, maximizing resource efficiency and reducing production costs [26].

When developing biobased and biodegradable packaging, it is also important to consider the method for evaluating the environmental impact of the material throughout its life cycle, from raw material extraction to disposal, termed LCA [10]. This assessment includes gathering input and output data, collected from all the process stages, from the collection of the raw material, processing, manufacturing, packaging, use, and maintenance to the disposal or recycling to understand the environmental footprint and identify potential areas for improvement in terms of resource use, energy consumption, greenhouse gas emissions, and waste generation [10,27].

## 3. Biobased and Biodegradable Polymers

**Figure 3:**
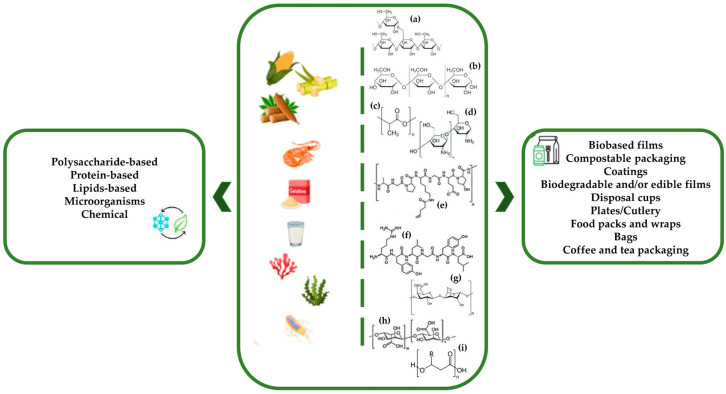
Biopolymers’ origin, chemical structures ((**a**) starch, (**b**) cellulose, (**c**) PLA, (**d**) chitosan, (**e**) gelatine, (**f**) casein, (**g**) carrageenan (k-carrageenan), (**h**) alginate, and (**i**) PHA), and their applications in the food packaging sector [38,39,40,41,42]. Created with Canva.com.

The environmental impact of using non-renewable plastic materials for packaging, particularly in food packaging, has underscored the urgency for sustainable alternatives [28]. However, in the process of finding these new alternatives, other issues have arisen. For instance, conventional petroleum-based plastics have higher performance when compared with biopolymer-based films. Polyethylene (PE) typically exhibits tensile strength (TS) values of 10–30 MPa and low water vapor transmission rates (WVTR superior to 4 g/m^2^·day), while polyethylene terephthalate (PET) combines higher strength (approximately 55–79 MPa) and interesting moisture barrier properties [29,30,31]. Starch-based films show wide variability in TS (0.36–40 MPa) and WVTR values (≈7.8–9 g/m^2^·day), which reflects their hydrophilic nature [32,33]. Cellulose and chitosan films can reach TS comparable to PE (13–100 MPa and 38–77 MPa, respectively), yet their moisture barrier performance remains inconsistent, particularly for chitosan (WVTR up to 145 g/m^2^·day) [32,34,35]. Polylactic acid (PLA) films exhibit TS close to PET (approximately 39.8–44 MPa) but significantly higher WVTR values (27–50 g/m^2^·day) [5,32,36]. Overall, these data show us that while several biopolymers approach conventional plastics in mechanical performance, inferior water vapor barrier properties remain the main competitive disadvantage. Moreover, most raw materials used for manufacturing biobased plastics still come from first-generation feedstocks (corn, wheat, potatoes, and sugarcane), and the use of second-generation feedstocks (lignocellulose) is still compromised regarding pre-treatment, hydrolysis, and downstream technology [37]. Another important challenge is related to the recycling industries associated with undesirable components in reprocessed products, which can compromise durability and strength. Also, conventional separation techniques struggle to differentiate between traditional and biobased plastics due to their identical densities, and current technologies like near-infrared spectroscopy face economic and technical problems [37]. Despite all these challenges, the development of new materials and packaging has brought us into the era of bioplastics, particularly biobased and biodegradable materials, with increased use in the food packaging sector. These materials may originate from either natural or synthetic sources, encompassing biomass, microorganisms, and chemicals as the three primary extraction sources [37], as summarized by Figure 3.

All the examples below share common characteristics such as biodegradability, renewability, and non-toxicity. Table 1 provides an overview of commonly used biopolymers in food packaging, summarizing their film type, positive and negative characteristics, and representative physico-mechanical properties.

### 3.1. Natural Origin

#### 3.1.1. Starch

Starch is a polysaccharide that consists of two macromolecules, amylose and amylopectin, as presented in Figure 3, and can be used in the food packaging industry as film or coating [4]. It is a natural polymer with abundant reserves, edibility, and reduced price [5,43]. However, organic starch itself has major limitations, such as brittleness and high hydrophily, that will influence the water resistance, hydrophobicity, and mechanical properties in wet environments [4,5,44]. In the food packaging industry, starch is mainly used to produce disposable cups, plates, cutlery, and food packs, and there are diverse companies already manufacturing starch-based films [5].

#### 3.1.2. Cellulose

Cellulose is a semicrystalline polysaccharide comprising repeated glucose units linked by β-(1–4) glycoside linkages with three –OH groups. Starch is very abundant and is a low-cost material [4]. The use of cellulose in food packaging exhibits positive characteristics, including being biocompatible, having good mechanical and physical properties, a good UV barrier, and high thermal resistance. Nevertheless, the use of cellulose for food packaging has some limitations, such as high-water absorption, brittleness, and weak interfacial adhesion [4,45]. Despite this, cellulose is already used to produce bags, wraps for food, films, as well as coffee and tea packaging, compostable packaging including snack bags and stick packs, and packaging for dry foods and bakeries [5].

#### 3.1.3. Chitosan

Chitosan is a deacetylated derivative of chitin, a linear polysaccharide that consists of D-glucosamine and N-acetyl-D-glucosamine linked with a β-(1 → 4) glycoside bond. It can be found in crustacean exoskeletons, insects, and fungi and is the second most abundant biopolymer after cellulose. Chitosan is a functional, versatile biopolymer due to the presence of amino groups responsible for the diverse properties of the polymer. It is different from the other polysaccharides due to its nitrogen content. Chitosan exhibits high biocompatibility, solubility, viscosity, ion binding, film-forming ability, and good antimicrobial and antioxidant activity [46,47,48]. These characteristics make it an excellent option for different industries, such as cosmetics, medicine, agriculture, but principally in the food industry, which is used to produce biodegradable films and edible coatings [49]. However, chitosan also has considerable limitations, such as high sensitivity to water, low mechanical and thermal stability, and susceptibility to brittleness under certain conditions [48].

#### 3.1.4. Alginate

Alginate is a natural polymer mainly isolated from the cell walls of brown algae (*Phaeophycean* spp.), where it is found in the form of sodium, calcium, and magnesium salts of alginic acid, but can also be synthesized by bacteria, for example, *Pseudomonas* and *Azotobacter* [50,51]. Alginate has some interesting properties for the food packaging sector, such as biocompatibility, film-forming, low permeability to O_2_ and vapours, good tensile strength, flexibility, tear resistance, rigidity, water solubility, and gloss while being tasteless and odourless [4,51]. These characteristics make alginate a good alternative to form films and coatings [52]. Nonetheless, alginate also presents limitations, like brittleness and high hydrophobicity, which will influence the water vapor transmission rate, as well as a poor moisture barrier, susceptibility to UV radiation, and sensitivity to microbial growth [4,51].

#### 3.1.5. Carrageenan

Carrageenan is a natural linear sulphated polysaccharide that is refined from red edible seaweeds such as *Chondrus crispus*, which are the most popular red edible seaweeds used to produce carrageenan. Carrageenan is widely used in the food industry because of its biocompatibility, high gel-forming ability, thickening, and stabilizing properties, but also due to its protective coating, fat substitution capabilities, and antibacterial properties [4,53]. Furthermore, it is used in the food packaging industry to produce films, but also food containers and cups [5]. Some limitations include hydrophilicity, which will influence the water vapor permeability and resistance, and poor mechanical properties [4,53].

#### 3.1.6. Soy Protein

Soy protein is a plant protein commonly used in the food industry due to its functional properties and nutritional value. This material has the potential to be suitable as a food packaging material, to be produced as edible and/or biodegradable film, due to its abundance and sustainability, strong biocompatibility, and being a low-cost material [52]. Nevertheless, there are some disadvantages associated with soy protein, for example, reduced water tolerance, low mechanical and thermal resistance, and processing limitations [4,54].

#### 3.1.7. Zein

Zein is a protein found in the endosperm of maize, is a by-product of the starch production process, and has high solubility in ethanol and high insolubility in water. Because of these characteristics, zein-based films exhibit good barrier properties to moisture and are reused for the packaging of foods that are sensitive to moisture, such as nuts and patisserie products [4]. This polymer is also used for coating food containers due to its resistance to being penetrated by oils and greases [52]. On the other hand, zein also has limitations, like brittleness and poor mechanical properties [4].

#### 3.1.8. Casein

Casein is a phosphoprotein and exhibits many advantages, such as high nutritional value and biocompatibility, the capability of forming a gel, emulsification, foam production, water absorption, and very good stability [4]. In the manufacturing industry, it is used as a binding agent, but it is also great to make edible films [55]. However, caseins also present some considerable disadvantages, such as low mechanical properties and poor barrier properties, especially to moisture, gases, and volatile compounds [4].

#### 3.1.9. Whey Proteins

Whey proteins are obtained from whey, the liquid phase created after cheese production. This sub-product can be used as a biopolymer source and turned into edible films and/or coatings [56]. This has some positive characteristics, such as high elasticity, good barrier properties, and film-forming ability. Nevertheless, it has its limitations, like low moisture sensitivity and mechanical properties [4].

#### 3.1.10. Gelatine

Gelatine is a peptide produced by the partial hydrolysis of collagen. The predominant physicochemical property of gelatine is its capacity to form gels. Furthermore, gelatine is a low-cost, abundant material that has high elastic abilities and is a good stabilizer, emulsifier, foaming, and micro-encapsulating agent [4]. Gelatine is an essential food additive, and the gelatine-based films and coatings have high mechanical and functional properties [56]. The main limitation of these films is the poor water barrier properties [4].

#### 3.1.11. Glycerides and Waxes

Glycerides and waxes have as predominant characteristics high insolubility to polar solvents and high solubility to non-polar solvents. The films produced are used as coatings in biodegradable films that have high hydrophilicity, including films made by proteins and polysaccharides. Lipids possess good moisture barrier properties and water resistance due to their hydrophobic nature. On the other hand, lipid-based films/coatings have low physical properties [4,57].

#### 3.1.12. Polyhydroxyalkanoates (PHA)

PHA is a varied range of polymers, including polyhydroxybutyrate (PHB), poly(3-hydroxybutyrate-co-3-hydroxyvalerate (PHBV), polyhydroxyvalerate (PHV), and polyhydroxyheptanoate (PHH), produced by bacterial fermentation [58]. Food waste materials such as fats, domestic waste, frying oil, crude glycerol, starch, fructose, maltose, and xylose can be used to produce these polymers. PHAs possess high biodegradability and similar properties to conventional plastics such as PE and polypropylene (PP). Nevertheless, PHA packages also have disadvantages such as high brittleness, high thermal sensitivity, limited malleability, and high permeability to gases [59]. Additionally, this polymer can be used to produce hot/cold cups, cup lids, yogurt containers, tubs, trays, and single-serve food packaging, and is considered a potential substitute for aluminium foil [60].

**Table 1:** Overview of biopolymer packaging films, including film type, key characteristics, reported physico-mechanical properties, such as TS, elongation break (EB), and WVTR or water vapor permeability (WVP), and current limitations of the biopolymer if used in their basic form.

Biopolymer	Film Type	Physico-Mechanical Properties	Characteristics	Current Limitations	References
Starch	Polysaccharide-based	TS (MPa): 0.36–40 EB (%): 27–137 WVTR: 7.8–9 g/m^2^/d; 4.60–4.67 mg/cm^2^.h WVP (g.mm/m^2^.day.kPa): 5–10	Abundant Edible Low price Antimicrobial and antioxidant activities Film-forming capabilities Easy processing	High water vapor permeability, moisture sensitivity, brittle without plasticizers, retrogradation over time reduces mechanical and barrier properties, and limited thermal stability.	[4,5,32,33,34,43,44]
Cellulose	Polysaccharide-based	TS (MPa): 13–100 EB (%): 4–10 WVTR (g/m^2^/d): 4.6–9 WVP (g.mm/m^2^.day.kPa): 1–5	Biocompatible UV barrier Insufficient interfacial adhesion	Limited solubility and processability, requiring modification for film formation, the hydrophilic nature leads to poor water vapor barrier properties, along with limited mechanical strength and transparency.	[4,5,32,34,45]
Chitosan	Polysaccharide-based	TS (MPa): 38–77 EB (%): 17–76 WVTR (g/m^2^/d): 0.5–145.3	Abundant High biocompatibility Soluble Viscosity Ion binding Film-forming ability Antimicrobial and antioxidant activity	Hydrophilic, moisture sensitivity, brittle without plasticizers, moderate water barrier but good gas barrier, functionality affected by pH, requires modification or composites for industrial use.	[5,32,35,46,47,48,49,61]
Alginate	Polysaccharide-based	TS (MPa): 30–50 EB (%): 3.8 WVP (g.mm/m^2^.day.kPa): 3–7	Biocompatible Gel properties and film-forming ability Low permeability to O2 and vapours Water solubility Gloss Tasteless and odourless Susceptible to UV radiation Sensible to microbial growth	Poor moisture barrier and high hygroscopicity, brittle structure, low water resistance, and dissolves rapidly in water at room temperature.	[4,5,34,50,51,52]
Carrageenan	Polysaccharide-based	TS (MPa): 24.73–65.20 EB (%): 10.75 WVP: 0.383 g.mm/m^2^.h.kPa/1.89 × 10^−11^ g.m.Pa^−1^.s^−1^	Biocompatible Gel-forming ability Thickening and stabilizing properties Protective coating Fat substitution capabilities Antimicrobial properties	Limited application due to poor mechanical strength and low water resistance, leads to brittleness and instability under humid or aqueous conditions.	[4,5,53,62,63]
Soy Protein	Protein-based	TS (MPa): 5–15 WVP (g.mm/m^2^.day.kPa): 2–10	Abundant Sustainable Biocompatible Low cost Low water resistance Low strength	Poor water resistance, low thermoplasticity, and brittleness, resulting in low TS and limited mechanical performance without modification.	[4,35,52,54]
Zein	Protein-based	TS (MPa): 3.1–4.2 WVP (g/m.s.PA): 420 × 10^−10^	Good barrier properties to moisture High solubility in ethanol High insolubility in water	High brittleness, poor processability, with limited mechanical and thermal properties.	[4,30,52,64]
Casein	Protein-based	TS (MPa): 1.39 EB (%):4.97 WVP (g.mm.m^2^.h^1^.kPa^1^): 0.673	High nutritional value Biocompatible Gelation Emulsification Foaming Water-binding ability Stable	Brittle, low water resistance, poor mechanical strength, and limited processability, requires plasticization or blending for functional films.	[4,55,65]
Whey proteins	Protein-based	TS (MPa): 5–12 WVP (g.mm/m^2^.day.kPa): 1–2	High elasticity Film-forming ability	Poor TS and moisture resistance restrict its application in high-humidity food packaging.	[4,35,55]
Gelatine	Protein-based	TS (MPa): 17 EB (%): 20 WVTR (g/m^2^/d): 290 WVP (g.mm/m^2^.day.kPa): 1–3	Low cost Abundant High elastic abilities Acts as a stabilizer Emulsifier Foaming and micro-encapsulating agent Gel forming	Poor mechanical properties and limited processability make it unsuitable as a standalone food packaging material.	[4,32,55]
Glycerides and Waxes	Lipids-based	TS (MPa): <5 WVP (g.mm/m^2^.day.kPa): 0.05–1	High insolubility in polar solvents High solubility in non-polar solvents Hydrophobic nature Good moisture barrier properties Water resistance	Hydrophobic barrier materials with low mechanical strength and flexibility, limited structural stability when used alone	[4,35,57]
PHAs	Microorganisms	TS (MPa): 20–40 (PHBV); 25 (PHB) EB (%): 2.3 (PHBV); 5 (PHB) WVTR (g/m^2^/d): 10 (PHBV); 1.16 (PHB)	Biocompatible Tailorable properties depending on the monomer unit Fully biodegradable in several different natural conditions and environments Excessive carbon Limited nitrogen sources Brittleness Thermal sensitivity Limited malleability High gas permeability	Moderate barrier properties, requires blending for improved mechanical performance, high production cost limits large-scale and cost-competitive use.	[30,32,58,59]
PLA	Chemical	TS (MPa): 39.8–44 EB (%): 2.7–30.7 WVTR (g/m^2^/d): 27–50	Mechanical resistance High saleability at low temperatures Effective barrier against flavour and odour Reduced energy consumption and carbon emissions Low waste production Brittleness Low heat-resistance capacity High production costs	High water vapor permeability, moderate oxygen barrier, brittleness, thermal sensitivity during processing, higher cost than conventional plastics.	[5,32,36,48,58,59]

### 3.2. Synthetic Origin

#### PLA

PLA is produced through the polymerization of lactic acid monomers. These monomers are obtained from the fermentation of starch or other carbohydrate-rich products, for example, wheat, corn, sugarcane, or kitchen waste. PLA presents several positive traits, including mechanical resistance, high seal ability even at low temperatures, an effective barrier of flavour and odour for food items, reduced level of energy consumption and carbon emissions, and low amounts of waste throughout the production process. However, the application of PLA in food packaging has some challenges, including the high brittleness of the material, weak gas barrier, low heat-resistance capacity, and relatively high production costs [48,58]. This material is mostly used to produce food packaging containers and foils, but also for disposable packaging like bottles, cold drink cups, containers with thermoformed trays and lids, overwrap packaging, and flexible films [60].

## 4. Improving Biobased Polymers with Bioactive Additives

The disadvantages related to the previous materials reviewed can be reduced by adding different additives, such as plasticizers, antimicrobial and antioxidant compounds, etc., making them good materials to replace traditional plastic packaging. There are already successful applications of biodegradable and compostable food packaging in the market, such as compostable food containers, coffee pods, and edible and biodegradable packaging films [5]. Moreover, incorporating natural extracts can make the packaging sector sustainable by repurposing waste materials that would otherwise be discarded, adding value to the products, and implementing the circular economy in the industry [66,67,68]. The main alternatives are PLA material, starch-based bioplastics, cellulose, chitosan, gelatine, and soy protein [60]. As previously mentioned, improving the suitability of biopolymers as alternatives to traditional plastics can be solved by incorporating additives, such as natural extracts with bioactive properties. Many biopolymers used in food packaging lack inherent antimicrobial activity or antioxidant ability; such properties can be conferred upon them through the incorporation of different agents, including nanoparticles and bioactive compounds [5].

**Figure 4:**
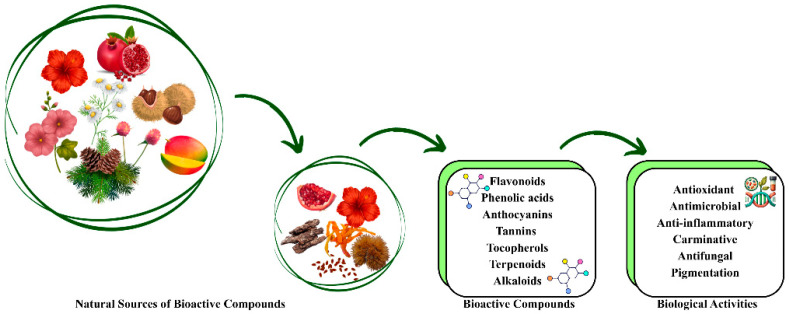
Nature-based bioactive compounds and their biological activities. Created with Canva.com.

Bioactive properties concern the attributes of packaging materials that interact with the food products themselves, potentially impacting their quality, safety, security, or shelf life. Such properties may require the release of specific compounds from the packaging material into the food or its ability to interact with food components, aiming to reinforce or inhibit specific biochemical reactions for food preservation. Among the most significant bioactive properties are antimicrobial activity, antioxidant capacity (oxygen scavenging), and moisture regulation [68], as represented in Figure 4. Antimicrobial activity is particularly important to prevent or reduce microbial growth, the biofilm or colonies formation on the food superficies especially related to food products with high water content, like meats, dairy, and fresh produce, making them very perishable. The product’s oxidation is also a major factor in food degradation, especially in products with high fat content, like nuts. Antioxidant activity is crucial to cease product oxidation and prevent the formation of free radicals, which can trigger chain reactions and compromise product quality [68]. Bioactive compounds such as polyphenols, which are abundant in agro-industrial by-products like grape pomace and olive leaves, are potent antioxidants. These polyphenols effectively halt oxidative processes, thereby preventing the deterioration of lipids and maintaining the food’s sensory properties. Furthermore, exposure to oxygen is a key contributor to both microbial spoilage and the degradation of food quality, affecting taste, odour, and colour [69]. Oxygen scavenging systems are particularly beneficial in packaging products like baked goods, cheese, and cured meats, where oxygen can significantly alter the product’s freshness over time [70]. Moisture regulation is a critical parameter in food packaging as excess moisture can lead to the growth of spoilage-causing microbes, while moisture loss can degrade the texture and quality of certain foods [71]. In dry foods such as cereals and snacks, moisture regulation is crucial for preserving texture, while in high-moisture foods like fresh fruits, it is key to avoiding spoilage [72]. Regarding the food packaging industry, incorporating natural extracts from agro-industrial by-products can offer numerous potential benefits. These extracts may add desirable physicochemical and functional properties to packaging materials, such as improved barrier properties, moisture resistance, and mechanical strength. These enhancements can improve the performance of packaging materials and ensure the integrity and safety of packaged foods throughout the food chain [73,74]. Natural extracts, rich in active compounds comprising phenolic compounds, terpenoids, flavonols, e.g., can function as natural antioxidants and antimicrobials while also enhancing food quality and extending the shelf life of the product by being incorporated into packaging material [75,76]. Various types of natural extracts are being used or studied for inclusion in the food packaging industry. For instance, green tea extract, as a non-fermented product, is an excellent source of polyphenolic compounds, particularly catechins, which have strong antioxidant and antibacterial properties [77,78]. Turmeric extract is used due to its broad-spectrum antifungal and antimicrobial activities; when incorporated into bio-based films, such as chitosan, it improves their mechanical properties [79]. Similarly, grapefruit seed extract is rich in flavonoids (naringenin), limonoids (limonin), ascorbic acid, and tocopherols, offering significant benefits [80,81]. In Table 2, some examples of natural extracts and agro-industrial by-products are presented.

## 5. Incorporation Techniques of Bioactive Compounds

**Figure 5:**
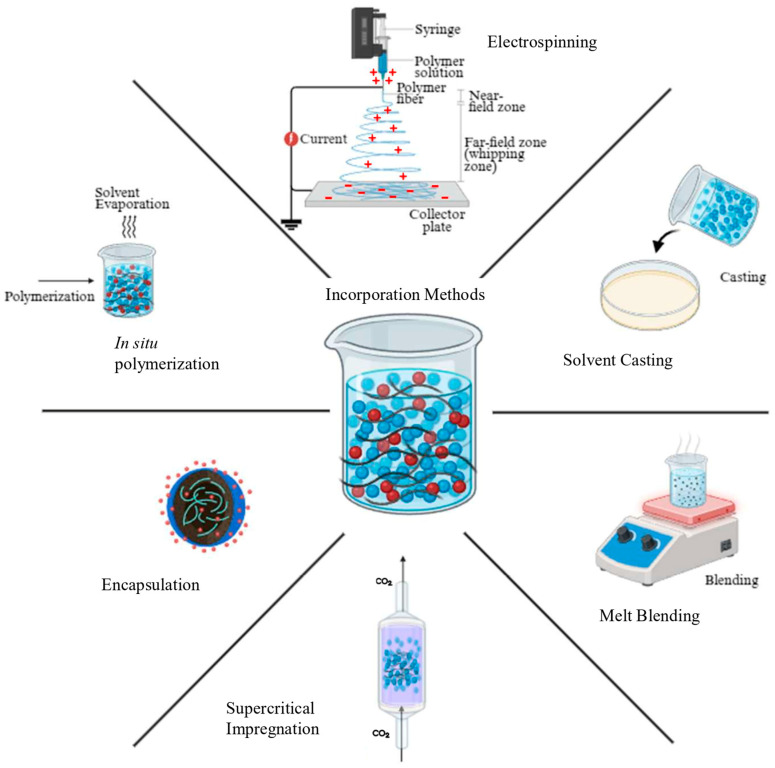
Incorporation techniques of bioactive compounds [125,126,127,128,129]. Created in BioRender.com.

The process of incorporating bioactive compounds involves their interaction with the biopolymer without the need for reactive side chain groups to bind. When adding natural extracts rich in bioactive compounds to films, the food to which the films are intended, the characteristics of the preferred packaging method, and the bioactive compound to be utilized are the most important factors to consider [48]. Therefore, it is understandable that the way the source of the bioactive compounds is processed (flour, pure extract, and essential oil) will also influence the production of the biodegradable packaging material. Despite the beneficial functionalities of the natural extracts, the substrate or the matrix has an important role in retaining the bioactive compounds through specific mechanical or physicochemical interactions. Implies some downsides to these extracts, like instability, a consequence of diverse environmental factors, such as temperature and pH variances, ultraviolet radiation, and oxidative agents [48]. Accordingly, various methods can be applied for integrating bioactive compounds from natural extracts into film-forming solutions made of biobased polymers, as synthesised in Figure 5.

Firstly, it is possible by solvent casting, which consists of casting the bioactive compounds with the biopolymers. By dissolving the polymer in an appropriate solvent, all while ensuring uniform dispersion, simultaneously with the active ingredient of interest. This is followed by transferring the mixture into a specific container or substrate for the solvent to be removed or evaporated in regulated conditions, producing a solid biopolymer composite embedded with the bioactive compound [125]. This technique brings advantages like improved dispersion and the possibility of hydrogen bonding with the polymer matrix, and promising mechanical, thermal, and biological properties, making it widely used in laboratory-scale studies, although it is limited by high time and energy consumption and low scalability. Likewise, it is possible to apply the melt blending technique to incorporate the bioactive component into molten polymeric material by extrusion, enhancing properties such as water barrier performance. It is better suited for industrial-scale applications but may cause thermal degradation for heat-sensitive compounds [48,66,67,125]. Electrospinning is another method used to produce nanofibers with high surface area, small inter-fibrous pore size, and high porosity by electrostatically spinning a solution containing the biopolymer and bioactive compounds into fine fibres. Moreover, electrospinning has been used to develop bi- and multilayer films for packaging applications, facilitating controlled release of bioactive compounds, and increasing thermal stability; however, it is generally restricted to laboratory-scale studies [125]. There is also the in-situ polymerization approach, which allows for exact control over the stability and dispersion of the bioactive component by chemically integrating it into the biopolymer matrix during the polymerization process. This method has already been used in different applications, including drug delivery systems and functional films, though requires careful reaction management [125]. Furthermore, there is the encapsulation method, which consists of retaining the bioactive compounds inside a wall material, forming micro or nanoparticles, and subsequently incorporating them by physical and direct mixing with the biopolymer in the film production or spraying them onto the biopolymer during the film processing. This technique improves solubility, enables controlled release, and facilitates targeted delivery while safeguarding bioactive molecules from degradation and extending their longevity [48,125]. Encapsulation includes different techniques, including emulsification-solvent evaporation, spray drying, and nanoprecipitation, that are important to ensure the stability and efficacy of the bioactive compound’s incorporation, supporting both laboratory and industrial-scale, but their effectiveness depends on the wall material, compound solubility, and process conditions [48,125]. Table 3 summarizes the incorporation techniques, including the type of incorporation, what it is based on, and the main advantages and limitations. In general, these methods highlight the trade-offs between processing complexity, material performance, and scalability, with melt blending and encapsulation techniques showing greater promise for high-throughput industrial applications, while solvent casting, electrospinning, and in-situ polymerization are more suitable for laboratory-scale studies.

## 6. Applications and Case Studies

Table 4 compiles examples of different biodegradable and bioactive packaging tested in diverse food products, illustrating the versatility of biobased polymers (coatings and films) and their potential for the food packaging industry. For instance, ongoing research shows that incorporating additives can significantly improve the functional properties of these materials. For example, the use of chitosan and beeswax for coating strawberries [130]. This improvement is partly attributed to hydrophobic interactions and physical integration of wax into the polymer matrix, which reduces water affinity and mobility [130,131]. Similarly, chitosan films incorporated with pomegranate peel extract, which has a high polyphenolic content, increase the film’s antimicrobial efficiency when applied to mangoes [132]. Another formulation blends chitosan with polyvinyl alcohol (PVA) and citric acid for coating strawberries and cherry tomatoes, resulting in improved mechanical strength, transparency, and antimicrobial activity. This behaviour is attributed to chitosan functional groups, especially amine and hydroxyl groups, which promote film formation, enhance solubility in acidic environments, and, when protonated, electrostatically interact with negatively charged microbial membranes, leading to membrane disruption and growth inhibition [133,134]. More recently, Liu et al. [135] developed a chitosan film incorporating *Tenebrio molitor* larvae protein and propolis ethanolic extract aimed at increasing the shelf-life of strawberries.

Beyond chitosan, starch-based films offer complementary benefits, composed of two polymers, amylose (linear) and amylopectin (branched). The interaction between amylose and amylopectin branches leads to an amorphous structure and a highly hydrophilic polymer, which limits the thermo-mechanical stability; nevertheless, these negative characteristics can be reduced [136]. For example, Lopes et al. [137] studied the use of starch films incorporated with phenolic extracts from potato peels, which increase antimicrobial and antioxidant activity, effectively prolonging the shelf-life of smoked fish fillets. Cruz-Galvez et al. [138] developed starch films using acetonic or methanolic extracts of *Hibiscus sabdariffa* for sausage packaging, which demonstrated significant antimicrobial activity, maintaining the quality and safety of the product. Panou and Karabagias [4] review several studies regarding starch derivatives used in combination with essential oils to create films for bakery products, but also other extracts for different food products, such as fruits, fish, and meat products. In the specific study of the essential oils, not only was there an improvement in antimicrobial properties, but also in the aroma and flavour profiles of the packaged goods. Zhou et al. [139] developed a biobased polymer film using a mixture of poly (butylene adipate-co-terephthalate) (PBAT) and nano-fibrillated cellulose, illustrating how interfacial engineering can enhance biopolymer performance. Additionally, the incorporation of a green surfactant additive (sucrose fatty acid ester) promoted dispersion and compatibility, resulting in improved mechanical and barrier properties. These improvements are attributed to strong hydrogen bonding between the ester groups of PBAT and cellulose nanofibrils hydroxyl groups, which strengthens interfacial adhesion, increasing tensile strength and reducing water permeability [140]. However, dispersion of cellulose nanofibrils can limit molecular-level interactions and decrease efficiency. This shows the importance of strategies that improve interfacial compatibility to achieve mechanically robust, high-barrier, and sustainably processable PBAT–cellulose composites [140]. This biobased polymer film was applied to mushrooms, a highly moisture-sensitive and perishable food product; these films effectively limit water uptake and microbial growth, preserving quality over extended storage [139]. These examples demonstrate that combining biopolymers with bioactive compounds or polymer blends can strategically improve mechanical, barrier, antimicrobial, and antioxidant properties, supporting the use of sustainable packaging alternatives in different food products.

**Table 4:** Examples of biodegradable and bioactive food packaging.

Matrix	Additive Extract	Package	Food Product	Reference
Agar (*Gracilaria vermiculophylla* extract)	Zinc oxide nanoparticles	Films	Smoked Salmon	[141]
Alginate	Aloe Vera and Frankincense oil	Films	Green capsicum	[4]
	Thymol		Apple slices	
Alginate	Pectin	Coating	Fresh-cut mango	[130]
Carboxymethylcellulose	Candelilla wax	Coatings	Pears	
Cassava starch, corn starch, gelatine	Beeswax	Coatings	Guava	
Chitosan	Beeswax	Coatings	Strawberries	
Chitosan and pullulan	Pomegranate peel extract		Mango	[132]
Chitosan/PVA	Citric acid	Films	Strawberry and Cherry tomato	[133]
Chitosan/*Tenebrio molitor larvae* protein	Propolis ethanol extract	Films	Strawberries	[135]
Chitosan/Whey protein	Cranberry/quince juice	Films	Fresh-cut turkey pieces	[130]
Corn Zein/Soy protein		Films	Olive oil	
Corn Zein/Wheat gluten		Films	Grape	
Fish gelatine/Orange peel pectin		Films	Cheese	
PBAT/nano fibrillate cellulose	Green surfactant	Films	Mushrooms	[139]
PBAT/TPS	Sorbate and benzoate	Films	Fresh rice noodles	[142]
Pectin	Honey	Coating	Apple, cantaloup melon, mango, pineapple	[130]
PHA	Flavonoid (phloretin)	Films	Apples	[143]
PLA	Ag-Cu nanoparticles and cinnamon oil	Films	Chicken meat	[144]
PLA/PBAT	Grape seed extract/Zinc Oxide Nanoparticles	Films	Fresh-cut vegetables	[145]
PLA/PBAT	Tea polyphenols	Films	Soy protein–based meat analogues	[146]
Soy Protein	Chicken feather keratin	Films	-	[147]
Starch	Phenolic extracts from potato peel	Films	Smoked fish fillets	[137]
Starch	Hibiscus sabdariffa extract	Films	Sausage	[138]
Starch and starch derivatives	Essential oils	Films	Bakery products	[4]
	Citric pectin and flour from feijoa peel		Apples	
	Maqui berry		Salmon	
	Eugenol		Pork	
	Rosehip		Chicken breast	
	Green tea		Sliced bacon	
Starch/PLA		Films	Cherry tomato	[4]
Whey protein	Furcellaran/Yerba mate and white tea extract	Edible films	Rennet-curd cheese	[130]
Xanthan gum	Pomegranate peel extract		Mango	[148]
Zein/Gelatine	Tea polyphenols	Films	Fresh-cut kiwi, banana, and avocado	[130]

## 7. Consumer Influence and European Strategies Supporting the Bioplastics Packaging Development

From a consumer perspective, key factors influence the product choice, such as price, material, and origin attributes, with preferences leaning towards biobased polymer options made from sugarcane, wood, and rice hulls and favouring 100% bioplastic content. It is also possible to differentiate the types of consumers based on their preferences. For instance, material-conscious consumers tend to prefer bio-based plastics derived from rice, straw, and other biomass-based materials [8]. In contrast, eco-conscious consumers prioritize environmental impact and appreciate bioplastics’ biodegradability or compostability features. Origin-conscious consumers show interest in locally sourced products and bioplastics made from locally produced raw materials [8]. Familiarity with green products and awareness of biomethane influence consumer attitudes toward bioplastics, while perception factors such as perceived value, risk, and effectiveness, as well as knowledge of bio-based concepts and their benefits, also play a significant role [8,149]. There is an intrinsic relationship between consumer behaviour and perspective with market trends. The consumer can push the change in the packaging industry, particularly in this situation, the major response was to increase the availability of substitute materials to traditional food packaging [9].

**Figure 6:**
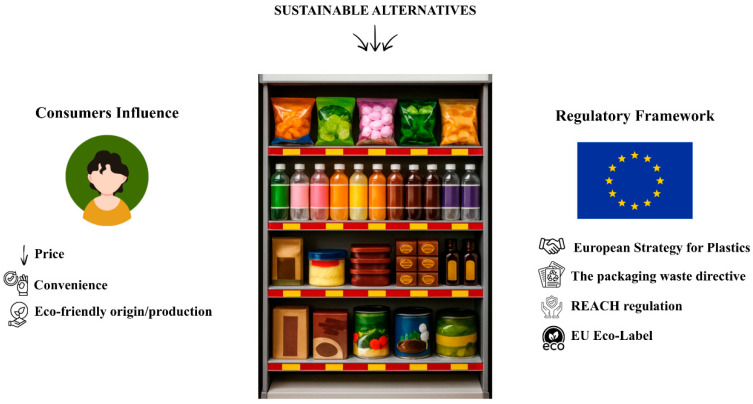
Consumers’ influence and European legal strategies towards food packaging sustainability. Created in Canva.com.

From the legal point of view, there are several regulations and guidelines within the European Union (EU) for companies developing and implementing biodegradable and bioactive packaging solutions to ensure environmental sustainability and consumer safety. The Packaging Waste Directive directs the member states to implement measures that prevent packaging waste while encouraging the use of eco-friendly packaging solutions [150]. Within this framework, EN 13432 serves as a harmonized standard, defining the requirements for composability and biodegradability of packaging materials to facilitate their recovery through natural degradation processes. Additionally, the REACH regulation ensures that chemicals used in the packaging follow the safety assessments, mitigating potential risks to human health and the environment [151]. To further promote sustainable products, the EU Eco-label certifies packaging made from recycled, biodegradable, or renewable materials, guiding consumers toward environmentally responsible choices. In alignment with these, the European Strategy for Plastics in a Circular Economy aims for all plastic packaging to be recyclable or reusable by 2030, raising innovation in sustainable material development [152]. However, biodegradable packaging incorporating antimicrobial properties is included in the Biocides Regulation, which is required to regulate the use of bioactive substances [153]. In addition to these regulatory frameworks, the use of bioactive compounds in food packaging introduces further legal complexity related to substance migration. When natural extracts are intentionally released from packaging and migrate into food to fulfil a technological function, such as antioxidant activity, they may be classified as food additives, thereby falling under Regulation (EC) No 1333/2008 [154]. This reclassification complicates regulatory approval, as the substance must undergo a full safety evaluation by the European Food Safety Authority (EFSA), comply with additive-specific purity criteria, be included in the EU positive list, and be declared on the food label. In contrast, substances that migrate unintentionally and do not exert a technological effect on food remain regulated under food contact materials legislation, Regulation (EC) No 1935/2004 and, for active and intelligent packaging, Regulation (EC) No 450/2009 [155,156]. Together, as represented in Figure 6, these regulatory measures ensure that biodegradable packaging meets high environmental and safety standards while supporting the transition to a circular economy [11,157]. Nevertheless, demonstrating or claiming functionality within the food represents a critical regulatory threshold, effectively shifting bioactive substances from packaging compliance into a more stringent and time-consuming food additive authorization pathway.

## 8. Future Perspectives and Challenges

**Figure 7:**
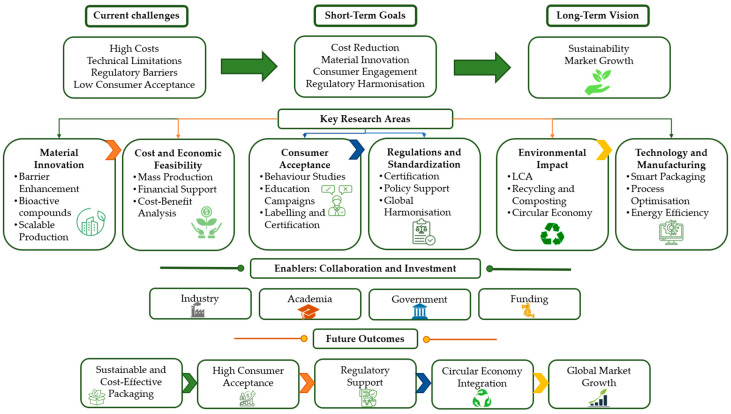
Representation of a Roadmap for Future Research.

Currently, it is already possible to find different biobased food packaging solutions in the market, mainly because of the Plastic Ban Law implemented in the EU, but also due to the increase in society’s environmental awareness and other pertinent factors. Still, it is necessary for further innovations, continued research in the field, collaboration across industries and academia, and a commitment to environmental management. The emerging packaging trends are usually linked to the effectiveness and positive benefits that they offer to the consumer and the possibility of choosing the better appropriate option. Biobased polymer packaging has been considered a feasible alternative to traditional packaging materials, aligning with the principles of the circular economy and enhancing the quality and safety of packaged food [66]. Nevertheless, this sector still needs a lot of improvement and research, the failure to include the packaging alternative in the market can be attributed to several factors, including low consumer acceptance of novel technologies, high costs, unresolved regulations, a lack of effective food product protection (such as moisture barriers), manufacturing challenges arising from material properties or sourcing issues, and competitive disadvantages [4]. To reduce production costs, one of the alternatives would possibly be mass production. Developments in technology facilitate scalable production of diverse packaging formats, meeting several food and packaging needs. The growing consumer demand for sustainable options drives investment and research in this field, yet challenges such as cost-effectiveness and regulatory compliance persist [158]. Also, the incorporation of bioactive properties into biodegradable packaging tackles environmental concerns and improves food preservation by extending shelf life and reducing food waste [158]. Despite the lack of preferential treatment for biobased packaging in Europe, certification proposals face obstacles due to uncertainty and high costs. Simplifying labelling and reducing certification gaps will be crucial for broader acceptance and long-term viability in the market [158]. Furthermore, it is imperative to assess the life cycle of these bioplastics and ensure that they are properly managed during disposal and treatment [4]. To achieve sustainable and visible results in a long-term approach, the relationship between environmental, economic, social, and regulatory factors needs to be enhanced and dynamized. To summarize the future perspectives and remaining challenges discussed in this review, Figure 7 provides a roadmap highlighting key research directions and priorities.

## 9. Conclusions

This narrative review highlights the potential for the application of biodegradable and biobased polymers in food packaging systems as a reliable pathway toward decreasing the environmental burden of conventional petroleum-based materials; nevertheless, their successful application remains conditional rather than inevitable. Notably, starch, cellulose, and PLA are already being incorporated in commercial packaging applications, while chitosan is gaining increased attention due to its favourable mechanical properties and intrinsic bioactivity, including antimicrobial and antioxidant activity. Recent studies have underscored the benefits of incorporating bioactive compounds into biopolymer matrices, which substantially enhance their mechanical strength, thermal stability, and barrier performance. Natural-derived bioactive compounds such as flavonoids, phenolic acids, and essential oils have demonstrated efficacy in prolonging food shelf life by inhibiting microbial growth and oxidative degradation. However, there is still the necessity to integrate material formulation or bioactivity enhancement and address adequate scalability, long-term stability, regulatory compliance, or real-world end-of-life scenarios. Insufficient standardization in life cycle assessment methodologies and limited data on migration behaviour and toxicological safety restrict meaningful comparison between biobased and conventional packaging systems. While PLA and starch-based packaging are already being produced on a scale, the costs of manufacturing more complex materials remain a barrier. Advances in processing methods, such as extrusion, multilayer structuring, and controlled-release systems for bioactive compounds, are critical to overcome current limitations related to moisture sensitivity, mechanical performance, and cost. Additionally, exploring second-generation feedstocks and improved extraction and purification methods for bioactive compounds can enhance both environmental performance and economic viability. There is a necessity that future research prioritises system-level optimisation rather than isolated material improvements. Beyond technological innovation, regulatory clarity and market alignment are decisive factors. Harmonised definitions and certification frameworks for biobased and biodegradable materials, including clear biodegradability and composability standards, are essential for industry and consumer trust. At the consumer level, targeted communication strategies are used in order to align environmental awareness with purchasing behaviour. Finally, regulatory policy, including financial incentives and support to end-of-life infrastructure, will play an important role in accelerating market acceptance. The regulatory policy should provide clear, harmonised guidance distinguishing food contact materials from food additives, particularly for bioactive packaging using natural extracts. Public funding and innovation incentives need to prioritize pilot-scale studies and technological and economic validation, while industry and academia collaboration is needed to define specific performance standards, such as acceptable WVTR, mechanical stability, and shelf-life extension thresholds. In conclusion, biobased and biodegradable food packaging should be viewed not only as a material solution, but as an evolving system requiring coordinated advances in materials science, safety assessment, market and consumer acceptance, and policy frameworks.

## Figures and Tables

**Table 2:** Sources of the natural extracts: type of extract, extracted origin, bioactive compounds, and biological activities.

Source	Type of Extract	Extracted From	Bioactive Compounds	Biological Activities	References
*Pinus pinaster*	Hydroethanolic	Bark Knot	Phenolic acids Flavonoids Stilbenes Lignans	Antioxidant Anti-inflammatory Antimicrobial Antifungal Anticarcinogenic	[82,83]
	Methanolic	Bark	Flavonoids Phenolic acids	Antioxidant Antimicrobial	[84]
*Taraxacum officinale*	Aqueous	Leaves Entire plant	Terpenoids Phenolic acids Flavonoids	Antioxidant Antibacterial Antiviral Anti-inflammatory	[85,86]
	Ethanolic	Leaves	Flavonoids Phenolic acids Terpenoids	Antibacterial Antioxidant Anti-inflammatory	[87]
*Hibiscus sabdariffa* L.	Hydroethanolic	Calyces Flowers and inflorescences	Phenolic acids Tocopherols Flavonoids	Antibacterial Antioxidant Antifungal Anti-inflammatory	[88,89]
	Aqueous	Calyces Flowers	Phenolic acids Flavonoids	Antiviral Antioxidant Colorant	[90,91]
*Peumus boldus* Molina	Hydroethanolic	Leaves Bark	Alkaloids Flavonoids Terpenoids	Anti-inflammatory Antifungal Antioxidant Trypanocidal activity	[92]
	Methanolic	Leaves	Alkaloids	Antioxidant Trypanocide activity	[92]
	Aqueous	Leaves	Alkaloids	Antioxidant Trypanocide activity	[92]
	Essential oils	Plant	Terpenoids	Anti-inflammatory Carminative Antifungal Antioxidant Antimicrobial	[92,93,94]
*Punica granatum* L.	Hydroethanolic	Fruit Peels	Flavonoids Tannins Coumarins Stilbenes	Antimicrobial Antioxidant	[95,96]
	Aqueous	Peels	Flavonoids Phenols	Antimicrobial Antioxidant	[97,98]
*Gomphrena globosa* L.	Methanolic	Leaves	Flavonoids Phenolic acids Betacyanin Tannins Phenols Tocopherols Terpenoids Alkaloids	Pigmentation Antioxidant Anti-inflammatory	[99]
	Aqueous	Flower/Plant	Flavonoids Phenols Phenolic acids Betacyanin	Pigmentation Antioxidant Antimicrobial	[100,101]
	Chloroformed	Leaves	Flavonoids Tannins Phenols Terpenoids Alkaloids	Antioxidant Anti-inflammatory	[99]
*Matricaria chamomilla* L.	Hydroethanolic	Leaves Flower	Phenolic acids Terpenoids	Antimicrobial Antioxidant	[102]
	Methanolic	Flower Tea	Flavonoids Phenolic acids Coumarin	Antioxidant	[103]
	Aqueous	Flower	Polyphenols	Antioxidant	[104]
*Malva sylvestris*	Hydroethanolic	Leaves Seed	Flavonoids Phenolic acids Quinones Terpenoids	Antioxidant Anti-inflammatory Anticarcinogenic Antimicrobial	[105,106]
	Aqueous	Petals	Flavonoids	Antioxidant;	[107]
*Castanea sativa*	Hydroethanolic	Peels Shells Burs	Flavonoids Phenolic acids Tannins	Antioxidant	[108,109]
	Methanolic	Peels Shells Burs Bark	Flavonoids Phenolic acids Tannins	Antioxidant Antimicrobial	[109,110,111]
	Aqueous	Peels Shells Burs	Flavonoids Phenolic acids Tannins Lignin	Antioxidant Antimicrobial	[108,109,112,113,114]
*Olea europaea* L.	Hydroethanolic	Olive Skin Stone Pulp Kernel Pomace	Flavonoids Phenolic acids Triterpenic acids Lignin	Antioxidant Anti-inflammatory Antibacterial	[115,116,117,118]
*Vitis vinifera*	Ethanolic	Peel Seed Stem Pulp Pomace	Flavonoids Phenolic acids Tannins	Antioxidant Antimicrobial	[119,120]
	Hydroethanolic	Peel Seed Stem Pulp Pomace	Flavonoids Phenolic acids Tannins Stilbenes Terpenoids	Antioxidant Antimicrobial Anti-inflammatory	[121]
*Mangifera indica* L.	Methanolic	Peels Seeds	Flavonoids Phenolic acids Tannins Carotenoids	Antioxidant Antimicrobial Anti-inflammatory	[122,123]
	Hydroethanolic	Peels	Flavonoids Phenolic acids Tannins Carotenoids	Antioxidant Antimicrobial Anti-inflammatory	[122,124]

**Table 3:** Overview of incorporation techniques of bioactive compounds.

Technique	Incorporation Type	Description	Advantages	Limitations	References
Solvent Casting	Blending	The bioactive compound is dissolved in a compatible solvent and mixed with the polymer matrix	Simple process Good dispersion Potential hydrogen bonding	Time and energy consumption Limited scalability	[125,126]
Electrospinning	Blending Surface Adsorption	Electrostatically spinning a polymer-bioactive solution into nanofibers using high voltage	High surface area Easy surface modification Cost-effective	Not suitable for large-scale applications	[126,127]
In-situ Polymerization	Covalent Bonding	During the polymerization process, the bioactive compound is chemically incorporated into the polymer matrix	Controlled distribution and stability of molecules	Requires control of reaction conditions	[126]
Encapsulation	Entrapment (Emulsification; Spray-drying; Nanoprecipitation)	Entrapping of the bioactive compounds within a wall material	Enhanced solubility Controlled/targeted release Protection of bioactive molecules	Sensitive to temperature Depends on the wall material and compound solubility	[126,128]
Supercritical Impregnation	Surface Adsorption Encapsulation	Deposition of the bioactive compound into the polymer matrix using supercritical fluids	Mild conditions Effective for hydrophobic molecules Solvent-free	High cost Limited to CO_2_- soluble compounds	[126,129]
Melt Blending	Blending	Direct mixing of bioactive compounds with polymers during melt processing	Good for organic and inorganic bioactive compounds Industrial scalability	High energy input Not suitable for heat-sensitive compounds	[126]

## Data Availability

No new data were created or analyzed in this study. Data sharing is not applicable to this article.
